# Preparation and Application of Apatite–TiO_2_ Composite Opacifier: Preventing Titanium Glaze Yellowing through Pre-Combination

**DOI:** 10.3390/ma17051056

**Published:** 2024-02-25

**Authors:** Xuefeng Bai, Han Zhang, Yu Tu, Sijia Sun, Yangzi Li, Hao Ding, Ming Bai, Liang Chang, Jianmeng Zhang

**Affiliations:** 1 Engineering Research Center of Ministry of Education for Geological Carbon Storage and Low Carbon Utilization of Resources, Beijing Key Laboratory of Materials Utilization of Nonmetallic Minerals and Solid Wastes, National Laboratory of Mineral Materials, School of Materials Science and Technology, China University of Geosciences, Beijing 100083, China; xfb980921@163.com (X.B.); zhanghan0050@163.com (H.Z.); 3003210007@email.cugb.edu.cn (Y.T.); 3003190001@cugb.edu.cn (Y.L.); 1003111107@email.cugb.edu.cn (M.B.); 2003140004@email.cugb.edu.cn (L.C.); 2103180006@email.cugb.edu.cn (J.Z.); 2 Beijing Building Materials Academy of Sciences Research Co., Ltd., Shixing Street, Shijingshan District, Beijing 100041, China

**Keywords:** apatite, TiO_2_, zirconium-free opacifier, sanitary ceramics, inhibiting glaze yellowing

## Abstract

In order to enhance the degree of binding reaction of TiO_2_ in titanium-containing ceramic glazes and prevent the reaction of its transformation into rutile to eliminate the yellowing phenomenon of the glaze surface, an apatite–TiO_2_ composite opacifier (ATO) was prepared through the mechanical grinding of hydroxyapatite and anatase TiO_2_. The properties, opacification mechanism, and yellowing inhibition of the prepared ceramic glazes were studied. The results show that the ATO is characterized by a uniform coating of TiO_2_ on the surface of the apatite and the formation of close chemical bonding between the apatite and TiO_2_. The ceramic glaze surface when using an ATO has a white appearance and excellent opacification performance. When an ATO was used, the L*, a*, and b* values of the glaze were 89.99, −0.85, and 3.37, respectively, which were comparable to those of a ZrSiO_4_ glaze (L*, a*, and b^*^ were 88.24, −0.02, and 2.29, respectively). The opacification of the glaze was slightly lower than that of the TiO_2_ glaze (L* value was 92.13), but the appearance changed from yellow to the white of the TiO_2_ glaze (b* value was 9.18). The ceramic glaze layer when using an ATO mainly consists of titanite, glass phase, and a small amount of quartz, and the opacification mechanism is the crystallization of the generated titanite. ATOs can play an active role in solving the critical problem that arises when TiO_2_ replaces ZrSiO_4_ as an opacifier.

## 1. Introduction

An opacifier is an inorganic material that generates opacified substances under high-temperature calcination conditions. A ceramic glaze added with an opacifier can show a strong opacifying effect after firing to cover the ceramic body, improve the function of the ceramic product, and provide a good appearance and aesthetic effect [1]. Common ceramic opacifiers include ZrSiO_4_ [2], SnO_2_ [3], ZnO [4], TiO_2_ [5], and phosphates [6,7]. Among them, ZrSiO_4_ has developed a stable application technology and production process for long-term use because of its stable opacification ability and the fact that it is not affected by a sintered atmosphere. It has become the most widely used opacifier in the ceramic industry [8,9,10,11]. However, in the processes of its production and application, zirconium silicate opacifiers have also exhibited some problems, such as the fact that the hardness of zircon sand as a raw material is very high (its Mohs hardness is 8~9) and the production costs of crushing and grinding are high. At the same time, zircon sand contains trace amounts of radioactive elements Th and U, resulting in certain radioactivity in zirconium silicate and ceramic products with zirconium silicate as an opacifier, which causes a threat to human health [12]. 

TiO_2_ is considered an alternative to ZrSiO_4_ as a ceramic opacifier due to its extremely high hiding power, refractive index, and non-radioactivity [13]. When TiO_2_ is used as an opacifier, TiO_2_ reacts with CaO and SiO_2_ during a high-temperature calcination process to form titanite (CaTiSiO_5_), which scatters visible light [14]. However, when TiO_2_ is singularly added to a glaze as an opacifier, TiO_2_ is often not fully involved in the formation of titanite. These free TiO_2_ particles are easily transformed into a rutile phase and cause the yellowing of the glaze, which has a negative effect on the appearance of ceramic products and is the main reason limiting the application of TiO_2_ as an opacifier. Using the solid-phase method, Li et al. [15] used anatase, calcium carbonate, and white carbon to prepare synthetic titanite powder. Preparing TiO_2_ in advance as a stable compound effectively inhibited the formation of rutile. Zhang [16] et al. synthesized perovskite by mixing a deactivated selective catalytic reduction (SCR) catalyst (mainly composed of TiO_2_) with CaCO_3_, and then perovskite was used as a titanium opacifier. The method of synthesizing perovskite through the pre-synthesis of TiO_2_ avoids TiO_2_ dissociating and a high-temperature phase transition to the rutile phase during ceramic sintering. Sun [14] et al. prepared a SiO_2_-CaCO_3_-TiO_2_ powder opacifier by wet grinding. Using the interfacial bonding between SiO_2_, CaCO_3_, and TiO_2_ can induce and accelerate the transformation of TiO_2_ to opaque titanite during the glaze sintering process, thus inhibiting the phase transition of anatase. These studies have shown that the pre-combination or pre-reaction between TiO_2_ and CaO or CaCO_3_ or CaO and SiO_2_ before the sintering of a glaze makes the synthesis of titanite a priority reaction in the sintering process, which is the key to improving the conversion degree of TiO_2_ to opacified titanite and inhibiting its conversion to rutile.

Phosphorus compounds are an important kind of chemical mineral raw material. In the ceramic industry, bone ceramics and bioactive glass ceramics are often produced by adding phosphorus compounds such as calcium phosphate [17], apatite [18], and bone ash [19] to enhance the appearance of ceramic products. Among them, apatite is divided into fluorapatite, chlorapatite, and hydroxyapatite according to its different binding anions [20]. The main components are CaO and P_2_O_5_, which play an essential role in the sintering of ceramic glaze. When apatite provides a CaO component, the precipitated P_2_O_5_ component can catalyze phase separation crystallization, making the crystal form faster, thereby improving the reflectivity of the glaze surface and making the glaze surface glossier. At the same time, a spherical glass differential phase is formed in the glaze, and the tiny glass phase separation close to the light wavelength leads to light scattering, thus having an opacifying effect [21,22].

Therefore, to improve and enhance the performance of TiO_2_ as a ceramic opacifier, solve the problem of glaze yellowing in its application process, and play the role of phosphorus compounds, in this study, hydroxyapatite was used as the carrier, and composite particles (ATO) coated with TiO_2_ on the surface of hydroxyapatite were prepared by a mechanical grinding method. In order to verify the opacifiers prepared from different sources of apatite, hydroxyapatite was replaced with concentrated apatite to prepare the opacifier (PTO). Different kinds of apatite were used as components to provide phosphorus compounds and were combined with TiO_2_ in advance to induce the reaction of CaO in TiO_2_ combined with hydroxyapatite and SiO_2_ in the glaze to form CaTiSiO_5_ so that TiO_2_ could form titanite without the formation of a rutile phase, to prevent the yellowing of the glaze induced by rutile in the glaze layer.

## 2. Materials and Methods

### 2.1. Materials

The raw materials used in this experiment are TiO_2_ and apatite. Among them, the TiO_2_ raw material is an anatase titanium dioxide product produced by Henan Lomon Billions Group Co., Ltd. (Jiaozuo, China). The XRD spectrum (in Section 3.1.2) shows that the characteristic peak of the anatase phase is obvious, and there are no other miscellaneous peaks. The D50 (median diameter) of the TiO_2_ raw material is 9.42 μm, and the D90 is 27.35 μm, indicating a serious agglomeration phenomenon. The apatite raw materials include reagent hydroxyapatite (HAP) and concentrate apatite (CAP). HAP was provided by Xi’an Haoyuan Biotechnology Co., Ltd. (Xi’an, China), and its chemical composition was analyzed using the XRF test (Table 1). CaO and P_2_O_5_ were the main components of apatite, accounting for 98.72%, indicating its high purity. CAP is obtained from the flotation of phosphate rock (Fanshan, China). From the XRF quantitative analysis in Table 1, CAP is mainly composed of apatite (86%), calcite (8%), and mica (6%). The D50 and D90 of HAP were 61.84 μm and 140.86 μm, respectively. The D50 and D90 of CAP were 98.50 μm and 289.43 μm, respectively. Both had large particle sizes, and further treatment was needed before compositing. The other reagents used in the test were mainly deionized water (self-made in the laboratory) and ethanol (Aladdin Reagent (Shanghai) Co., Ltd. (Shanghai, China)).

### 2.2. Preparation of ATO and Sanitary Ceramic Glaze

The preparation process for the ATO composite opacifier was as follows: (1) HAP was ground and disaggregated in a grinding machine (GSDM-S3, Beijing Gosdel & Technology Co., Ltd., Beijing, China. The grinding medium is a zirconia ball with a diameter of 1–3 mm; the grinding jar is made of polytetrapluroethylene material) to make its particle size meet the requirements of the ATO. The grinding conditions were as follows: the rotational speed of the grinding machine was 1000 r/min, the ball-to-powder ratio was 4:1, grinding was carried out for 60 min, and the solid content was 30%. After grinding, the D50 of HAP decreased to 1.92 μm, the D90 decreased to 4.49 μm, and the grinding effect was remarkable. (2) The TiO_2_ raw material was placed in the above grinding machine to grind and disaggregate. The grinding conditions were as follows: the rotational speed of the grinding machine was 1000 r/min, the ball-to-powder ratio was 4:1, grinding was carried out for 40 min, and the solid content was 30%. After grinding, the D50 of TiO_2_ decreased to 1.03 μm, the D90 decreased to 2.01 μm, and HAP and TiO_2_ reached the best particle size ratio of the particle composite. (3) The ground HAP and TiO_2_ were mixed in proportion and then mechanically ground to initiate the reaction between the two at the phase interface to obtain the apatite –TiO_2_ composite opacifier (ATO). The grinding conditions were as follows: mill speed was 1000 r/min, ball-to-material ratio was 4:1, grinding was carried out for 60 min, and the solid content was 30%. The samples with different TiO_2_ composite ratios were recorded as ATO-B, where B is the mass fraction of TiO_2_.

Ceramic samples with an ATO were prepared in an industrial roller kiln in a sanitary ceramics factory (Tangshan Zhongtao Sanitary Bath Manufacturing Co., Ltd., Tangshan, China), and the highest firing temperature was 1180 °C. The formula and proportion of the glaze are as follows (weight, %): potash feldspar, 28; quartz, 34; Suzhou kaolin, 4.5; alumina, 3; calcite, 11; dolomite, 5.5; zinc oxide, 2.5; frit, 2; methyl-rich cellulose, 0.15; CA-100, 0.07; opacifier, 8.5. (All raw materials in the glaze formula are from Tangshan Zhongtao Sanitary Bath Manufacturing Co., Ltd., Tangshan, China.). According to the above formula for an opacified glaze, the components of the basic glaze and the opacifier (with a total mass of 500 g) were weighed and placed in a ball mill, and then a 1000 g zirconium silicate grinding ball and 240 g of water were added to grind for 45 min. The grinding ball was separated by screening to obtain a glaze slurry, and the iron impurities were removed by stirring the glaze slurry continuously for 5~10 min with a magnet rod. Then, the standard porcelain body was glazed using the splashing glaze method, and the glaze thickness was about 0.8 mm. Finally, the glazed ceramic blocks were placed in an industrial roller kiln. To verify the opacifiers prepared from different sources of apatite, a PTO was prepared using the above grinding method with CAP and TiO_2_ as the raw materials, and the corresponding ceramic samples were sintered in a factory. For comparison, ceramic samples with pure TiO_2_ and ZrSiO_4_ as opacifiers were prepared using the same formula and sintering process.

### 2.3. Characterization

To characterize the opacifying effect of the opacifier, the Lab color values (L*, a*, and b*) and glossiness, which represent the glaze color, were tested. The color values (L*, a*, and b*) of the ceramic glazes were measured by an integrating sphere spectrophotometer (SP-60, X-Rite, Grand Rapids, MI, USA). The Lab color model is composed of brightness (L*), red–green (a*), and yellow–blue (b*) values. The L* value can be used to reflect the opacity performance of the glaze surface. The bigger the L* value of the glaze, the better the opacity performance. The a* and b* values can be used to calibrate the hue color of the glaze surface, where the b* value is positive, and the larger the glaze surface, the yellower it is. A gloss meter (WGG60-Y4, KSJ Photoelectrical Instruments Co., Ltd. (Shenzhen China) was used to measure the glossiness of the ceramic glaze. Glossiness is used to represent the degree of similarity of the surface of an object to a mirror surface. The measurement and evaluation of glossiness also depend on the angle between the test light source and the sample; in this paper, 60° is used for testing.

Characterization tests such as SEM, EDS, XRD, XRF, XPS, and FT-IR were used to observe the phase and microstructure and to study the bonding mechanism between HAP and TiO_2_. A scanning electron microscope (SEM, S-3500N, Hitachi Electron Microscope Co., Ltd., Tokyo, Japan) was used to observe the microstructure of ATO, the raw materials used, and the glaze layer of the ceramic samples. The observed glaze layer was first etched with 10 % HF to remove the glass phase. X-ray powder diffraction (XRD, D/MAX2000, Rigaku Corporation, Tokyo, Japan) was used to analyze and identify the phases in the samples. HAP and TiO_2_ opacifiers and an ATO were tested with an infrared spectrometer (Spectrum100, Shanghai Platinum Elmer Instrument Co., Ltd. (Shanghai, China)) and an X-ray photoelectron spectrometer (ESCAlab250, Thermo Scientific, Waltham, MA, USA) to explore the binding mechanism of HAP and TiO_2_.

## 3. Results

### 3.1. Morphology and Structure of ATO

#### 3.1.1. Effect of the TiO_2_ Ratio on the Morphology of ATO

**Figure 1:**
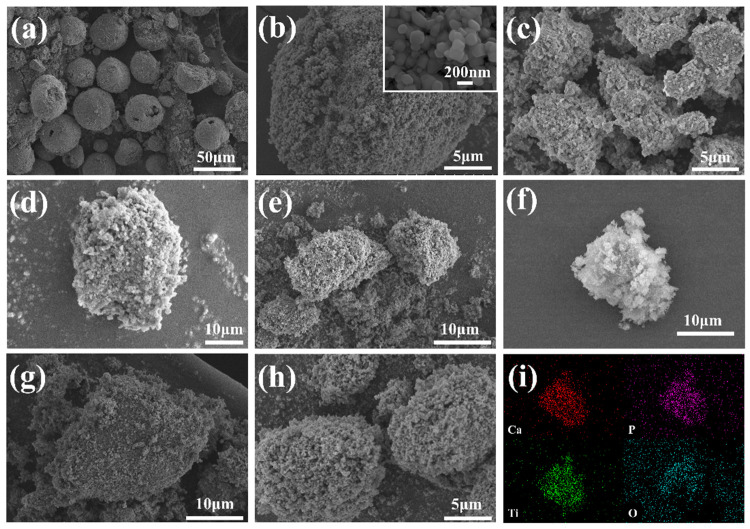
(**a**) HAP; (**b**) TiO_2_; (**c**) HAP grinding product; (**d**–**h**) ATO (30–40-50–60-70); and (**i**) EDS surface scan of ATO-50.

Figure 1 shows the SEM images of each sample. It can be seen from Figure 1a that the HAP raw materials are spherically distributed, the particles are large (about 50–60 μm), and the agglomeration is serious. The agglomeration of HAP after grinding (Figure 1c) is significantly improved, and the particle size is reduced to about 2–5 μm, which is more beneficial for its combination with TiO_2_. The morphology of the TiO_2_ raw material particles (Figure 1b) is spherical, and the particle size is between 200 and 300 nm, which is in the scale range of TiO_2_ pigment [23], but agglomeration occurs due to the small particle size. The HAP and TiO_2_ particles in the ATO composite particles are combined by mechanical force, which is characterized by a coating of TiO_2_ on the surface of the apatite to improve and reduce the agglomeration problem between TiO_2_ particles (Figure 1d–h). Among them, the ATO-50 sample has a better composite effect than the other samples. Figure 1i is the EDS scan of the ATO-50 sample. The Ca, P, O, and Ti elements are evenly distributed within the contour of the particles, indicating that TiO_2_ particles are more evenly distributed on the surface of the apatite and that the coating is more complete. To avoid the existence of free TiO_2_ in the ATO, ATO-50 was finally selected as the optimized composite condition.

#### 3.1.2. Phase Analysis of ATO

**Figure 2:**
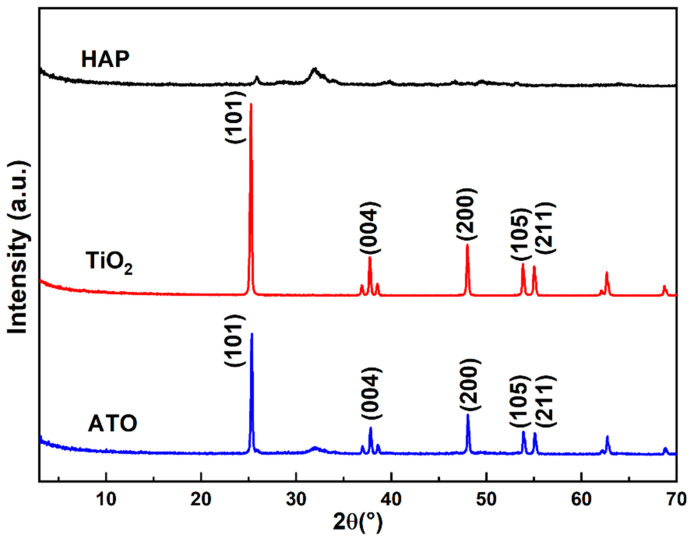
XRD spectrum of HAP, TiO_2_, and ATO composite opacifiers.

The phases of HAP, the TiO_2_ raw materials, and the ATO composite opacifier were analyzed using the XRD test method. From Figure 2, it is observed that the XRD spectrum of the raw hydroxyapatite has an approximately symmetrical and flat diffraction peak between diffraction angles (2θ) of 30° and 35°, and the main phase is HA. The five characteristic peaks in the XRD spectrum of TiO_2_ are located at 25.2°, 37.8°, 47.9°, 53.9°, and 55.1°, corresponding to the (101), (004), (200), (105), and (211) crystal planes of anatase (JCPDS 21-1272), respectively. The diffraction peaks are sharp, and there are no other peaks, indicating that the purity of the raw materials is very high. The XRD spectrum of the ATO has both the characteristic peaks of anatase and the characteristic peaks of apatite, and no new phase appears, demonstrating that the phase did not change during the composite process and that the correlation only occurs at the phase interface.

#### 3.1.3. Binding Properties between Apatite and TiO_2_

**Figure 3:**
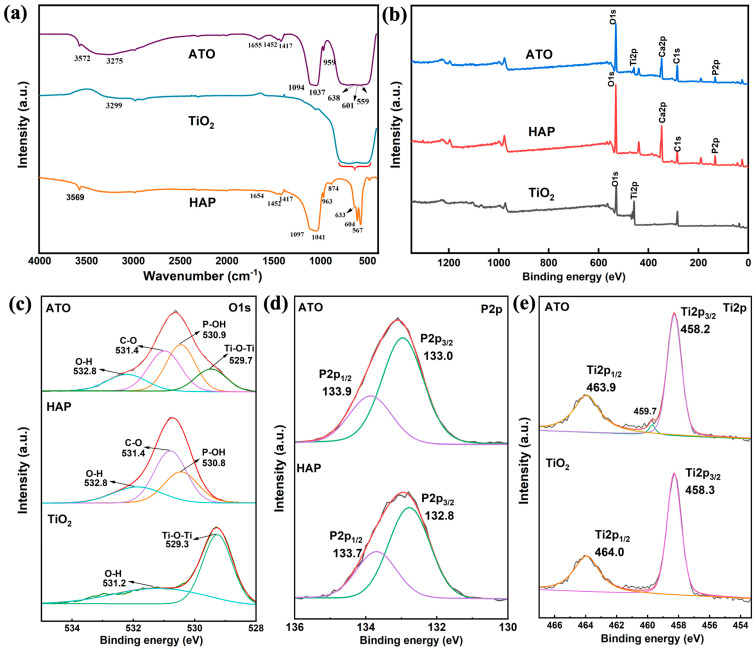
(**a**) FT-IR spectra of HAP, TiO_2_, and ATO composite opacifier and (**b**–**e**) XPS spectra.

To explore and analyze the binding properties and mechanisms of HAP and TiO_2_ in the ATO composite opacifier, the Fourier transform infrared spectroscopy (FT-IR) and X-ray photoelectron spectroscopy (XPS) of HAP, the TiO_2_ raw materials, and the ATO composite opacifier were tested. The results are shown in Figure 3. 

In the infrared spectrum of TiO_2_ in Figure 3a, the characteristic peak of the surface hydroxyl group appears at 3299 cm^−1^ [13], and the wide absorption band generated by the bending vibration of Ti-O-Ti is located in the range of 500–800 cm^−1^ [14]. In the infrared spectrum of HAP, the characteristic peaks at 567, 607, 963, 1041, and 1097 cm^−1^ belong to the vibration of the hydroxyapatite PO_4_^3−^ tetrahedron [24,25,26,27,28]. The absorption peak at 3569 cm^−1^ is related to -OH in HAP, and the absorption peak at 1649 cm^−1^ is related to the adsorbed H_2_O [29]. After the HAP and TiO_2_ composite, the wide absorption band of the Ti-O-Ti bond of the ATO sample appears at 500–1000 cm^−1^. At the same time, the characteristic peaks of PO_4_^3−^ shift to 559, 601, 959, 1037, and 1094 cm^−1^, respectively. The characteristic peak of the hydroxyl group on the surface of TiO_2_ moves to the low band and broadens. This indicates that the vibration mode of the P-O bond changed significantly after compounding with TiO_2_, indicating that HAP and TiO_2_ undergo chemical bonding on the surface. Through the obvious change in the hydroxyl position of HAP and TiO_2_, it is speculated that this process is completed by the hydroxyl action of the two. The detailed correspondence between the absorption peak and the functional group is shown in Table 2.

Figure 3b shows the characteristic peaks of O1s, Ti2p, Ca2p, C1s, and P2p that appeared on the XPS broad-spectrum scan of the ATO, which proved the existence of the five elements O, Ti, Ca, C, and P, reflecting its component characteristics. In the narrow scanning spectrum of O1s in Figure 3c, the binding energies of O1s in HAP are 532.8 eV, 531.4 eV, and 530.8 eV, which are related to the adsorption of water, CO_2_, and O atoms in P-OH, respectively [30,31]. From the narrow O1s scanning spectrum of ATO, the O atoms of P-OH, H-O-H, and C=O correspond to a binding energy of 530.9 eV, 532.8 eV, and 531.4 eV, respectively. Compared with the raw HAP, the binding energy of P-OH is reduced by 0.1 eV. In addition, according to Figure 3d, the binding energy of P2p in the ATO also changed greatly compared with the HAP, indicating that the chemical environment around the P atoms has changed. In the Ti2p scanning spectrum of the ATO, a peak different from TiO_2_ appeared at a binding energy of 459.7 eV [14], indicating that the binding energy of Ti2p1/2 and Ti2p3/2 derived from TiO_2_ also changed. It is speculated that HAP and TiO_2_ are combined by surface hydroxyl groups, resulting in changes in the binding energy of each element.

According to the analysis of the infrared and XPS spectra, combined with the surface properties of hydroxyapatite, the mechanism of HAP and TiO_2_ surface bonding can be analyzed. The P-OH group on the surface of HAP is the surface-OH group produced by the surface protonation of PO_4_^3−^ ions on the surface of HAP particles to maintain the balance of surface electrical properties [32,33]. Through the shift of the P-OH absorption peak in the FT-IR test results, the change in the P-OH binding energy in the XPS analysis, and the change in the chemical environment of the P and Ti atoms, it is speculated that there should be a combination of a P-OH group on the surface of the HAP particles and a hydroxyl group (Ti-OH) on the surface of TiO_2_ to form P-O-Ti so that the two are closely combined.

### 3.2. The Glaze Performance of Sanitary Ceramics with an ATO

#### 3.2.1. Effect of TiO_2_ Content in ATO on the Glaze Properties of Sanitary Ceramics

**Figure 4:**
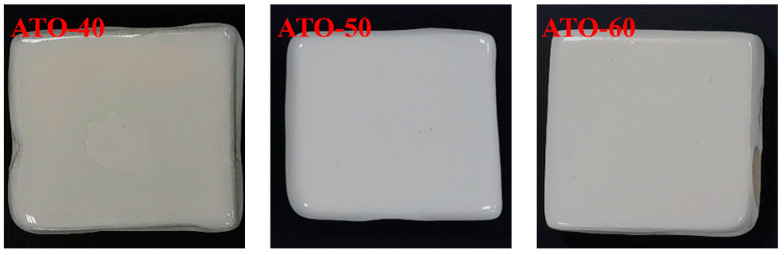
Digital photos of ceramic samples with ATO-40, ATO-50, and ATO-60 as opacifiers.

ATO-40, ATO-50, and ATO-60 were added to the ceramic base glaze as opacifiers to make a glaze slurry and sintered into a ceramic sample in a roller kiln. The Lab value, glossiness, and melting length of the glaze surface were tested. The test results are shown in Figure 4 and Table 3. As seen in Figure 4, when the opacifier is ATO-40, the ceramic glaze surface exposes the original color of the body due to insufficient covering ability (the L* value is only 74.10). When the content of TiO_2_ increases, the opacification performance of the glaze is also significantly improved, and a delicate, hard, and smooth white glaze is formed. When the content of TiO_2_ is 50%, the opacification performance of the glaze is the best, and the L* value is 89.99. When the content of TiO_2_ is too low, the precipitation of the opacifying phase is too small, the opacifying performance of the glaze is less, the glaze surface is affected by the body itself, and the color is dark. Compared with the sample with 50% TiO_2_ content, the appearance of the ceramic sample with 60% TiO_2_ content is yellowish, the L* value is 84.41, and the opacification performance is relatively poor. It was found that the b* value of the ceramic glaze with 60% TiO_2_ was 3.85, and the b* value of the glaze with 50% TiO_2_ was 3.37. Therefore, considering the appearance characteristics, opacification performance, and color of ceramic samples with ATOs, the ATO with a TiO_2_ content of 50% should be selected as the best opacifier.

#### 3.2.2. Comparison of Properties between Ceramic Glazes with ATO, PTO, ZrSiO_4_, and TiO_2_ as Opacifiers 

**Figure 5:**
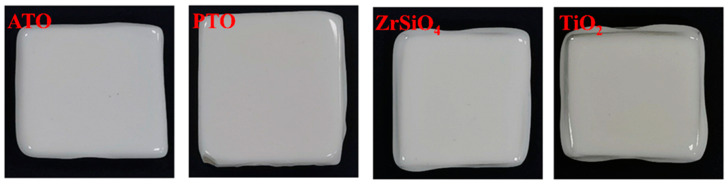
Digital photos of ATO, PTO, ZrSiO_4_, and TiO_2_ as opacifiers in ceramic samples.

Figure 5 and Table 4 are digital photos of ATO, PTO, ZrSiO_4_, and TiO_2_ as opacifier ceramic samples and data comparing the chromaticity, color, and characteristics of the glazes. The results show that the L* values of the ceramic samples with an ATO and PTO are 89.99 and 88.10, respectively, which are close to the L* value (88.24) of the ceramic samples with ZrSiO_4_ as an opacifier, indicating that the opacification properties of the ceramic samples using an ATO and PTO are comparable to those of ZrSiO_4_ opacifiers. The b* values of the two glazes are 3.37 and 3.10, respectively, which are similar to those of the glaze using ZrSiO_4_ (the b* value is 2.29), and the appearance of the three is white, indicating that using an ATO and PTO can achieve a similar effect as using an ZrSiO_4_ opacifier. In contrast, the b* value of the ceramic sample with pure TiO_2_ as the opacifier is much higher (the b* value is 9.18), and the color of the glaze is yellow. This result shows that an ATO or PTO can solve the problem of directly using TiO_2_ as an opacifier. This is attributed to the intense interfacial interaction between TiO_2_ and apatite or phosphate concentrate through mechanical force, which improves the degree of the TiO_2_ chemical reaction and inhibits the transformation of anatase to rutile during high-temperature sintering.

### 3.3. Mechanism of ATO

#### 3.3.1. Phase Analysis

**Figure 6:**
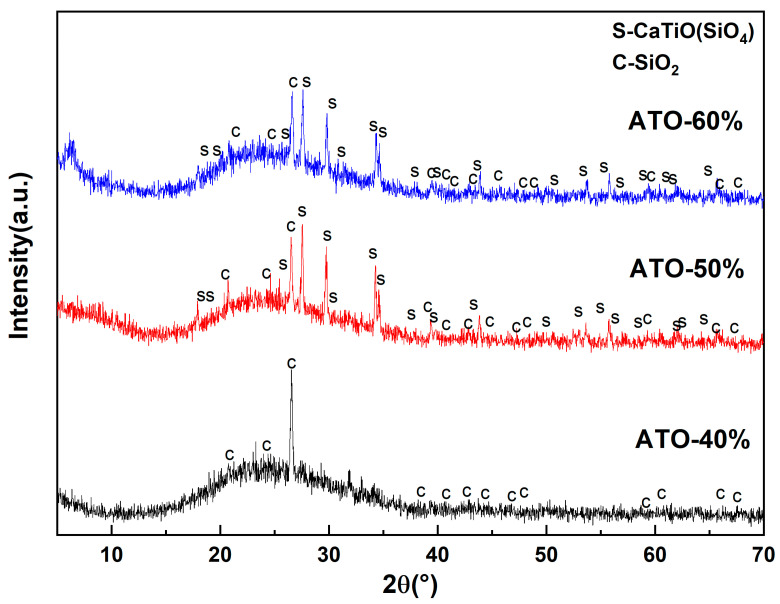
XRD spectrum of glazes with ATO-40, ATO-50, and ATO-60 as opacifiers.

To further explore the opacification mechanism of the ATO, the composition of the glaze layers of the ceramic samples with ATO-40, ATO-50, and ATO-60 were analyzed using an XRD test. The test resulted in Figure 6. Amorphous peaks appeared in the XRD spectra of the above three samples, indicating that there were many glass phases. In addition to sphene, there were obvious quartz peaks (C, SiO_2_) in the ATO-40 spectrum. Quartz and sphene phases (S, CaTiSiO_5_) were also found in the ATO-50 and ATO-60 spectra, and the amount of sphene phase in the ATO-50 spectrum was greater than that of the quartz phase, while no sphene phase appeared in the ATO-40 spectrum. According to the results of the glaze chromaticity values of ATO-40, ATO-50, and ATO-60, the content of titanite is closely related to the opacification performance of the glaze. The ATO-40 glaze does not generate titanite, so the opacification performance of the ATO-40 glaze is poor, while the high content of titanite in the ATO-50 glaze makes its glaze performance better. Therefore, it is considered that the formation of titanite during sintering is the reason for the opacification effect of the ATO, and quartz has an auxiliary opacification effect.

#### 3.3.2. Morphology Analysis

**Figure 7:**
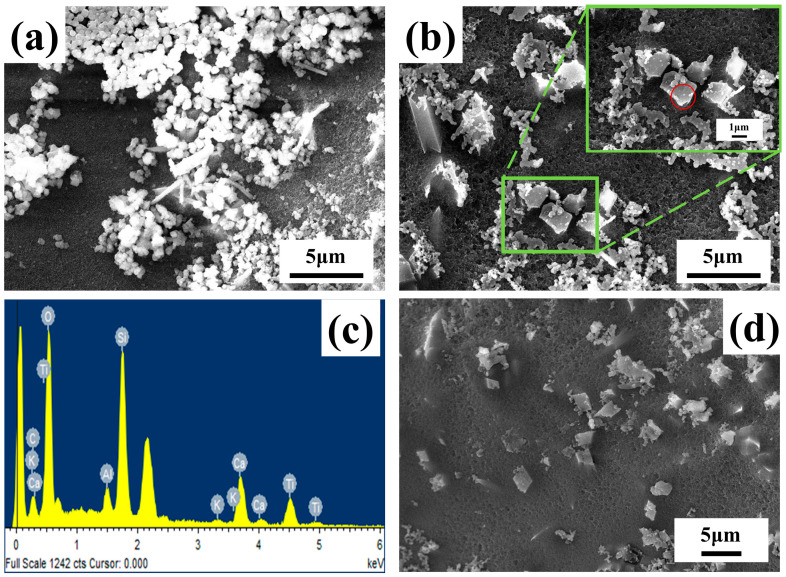
SEM images of (**a**) ATO-40, (**b**)ATO-50, (**d**) ATO-60 glaze surfaces after HF acid corrosion and (**c**) EDS element scanning in the red circle area in (**b**).

Figure 7 shows the analysis and characterization of the microstructure and morphology of the ATO composite opacifier glaze after HF acid corrosion by SEM. There are many spherical particles with a particle size of about 300 nm in the ATO-40 glaze layer, while the spherical particles in the ATO-50 and ATO-60 glaze layers become less, and wedge-shaped crystals with a particle size of about 2 μm appear. The EDS (Figure 7c) characterization shows that the wedge-shaped particles in Figure 7b are composed of Ti, Si, Ca, and O, and the atomic percentage of Ti, Si, Ca, and O is close to 1:1:1:5 (Table 5), which is the same as the chemical composition of titanite. Combined with the XRD test results and the characteristics of the titanite, it can be preliminarily inferred that the wedge crystal is titanite [16]. The spherical particles are amorphous, opaque particles composed of oxides such as Ca, Al, and Si. It should be that the phosphide generated by the decomposition of the apatite will induce the formation of a phase independent of the glass phase. According to the glaze performance data, the glaze covering ability of the ATO-50 and ATO-60 samples with titanite crystals is significantly stronger than that of the ATO-40 sample, which indicates that titanite is the component of the ATO agent with strong opacifying ability, while amorphous opacifying particles and quartz are auxiliary opacifying components.

**Figure 8:**
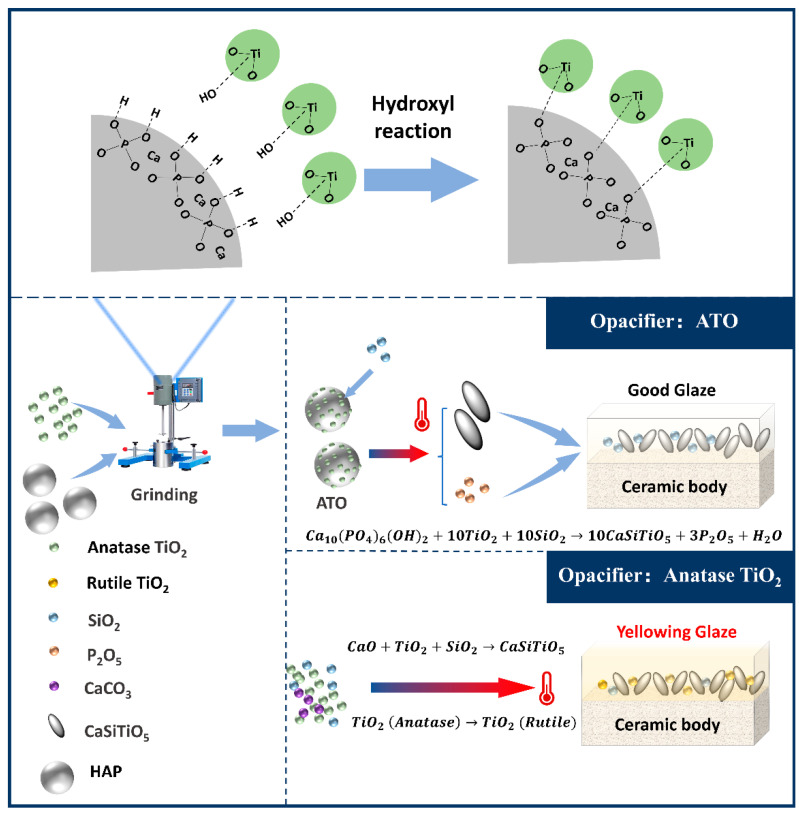
The mechanism of the composite ATO and the mechanism for the prevention of glaze yellowing.

Based on this, we can analyze the unique advantages of using an ATO instead of TiO_2_ as a ceramic opacifier. (1) The ordered combination of the interface between hydroxyapatite and TiO_2_ through the dehydration condensation of hydroxyl groups in advance creates the conditions for a preferential chemical reaction of TiO_2_ combined with CaO during the sintering process so that TiO_2_ can be fully incorporated into titanite, reducing the conversion of free TiO_2_ into the rutile phase during high-temperature firing and avoiding glaze yellowing. (2) After the decomposition of hydroxyapatite, the generated phosphorus oxide will induce the formation of a spherical glass differential phase in the glaze. These phases will lead to light scattering, which is more conducive to the opacification effect. The binding mechanism of HAP and TiO_2_ in the ATO and the mechanism of avoiding the yellowing of the titanium glaze with the ATO are shown in Figure 8.

## 4. Conclusions

The apatite–TiO_2_ composite opacifier (ATO) was prepared by mechanical grinding using hydroxyapatite (HAP) and anatase TiO_2_ as raw materials. The ATO is characterized by the uniform adhesion of the TiO_2_ on the surface of the apatite, and there is a close chemical bond between the HAP and the TiO_2_. White-glazed sanitary ceramics were prepared by using the ATO. The appearance of the ceramic glaze was white, and the opacification was excellent. The L*, a*, and b* values of the glaze were 89.99, −0.85, and 3.37, respectively. The opacification and whiteness of the glaze were similar to those of the ZrSiO_4_ glaze. The opacification of the glaze was slightly lower than that of the TiO_2_ glaze but whiter than TiO_2_. The composition of the glaze is a glass phase, titanite, and a small amount of quartz, in which titanite is in an opacified phase. The opacification mechanism is the crystallization opacification of the titanite phase, and the spherical glass formed at the same time is relatively beneficial to improving the opacification of the glaze surface. 

The advantage of this study is that when an ATO is sintered at a high temperature, the interfacial ordered bonding between hydroxyapatite and TiO_2_ induces the binding reaction of TiO_2_ with CaO and further with SiO_2_ at high temperatures so that TiO_2_ can enter the sphene without rutile phase formation, thus inhibiting the yellowing of the glaze. The use of an ATO can play an active role in solving the key problem that occurs when TiO_2_ replaces ZrSiO_4_ as an opacifier.

## Figures and Tables

**Table 1:** XRF results of HAP and CAP composites.

Types of Apatite	CaO	P_2_O_5_	SiO_2_	Fe_2_O_3_	MgO	Al_2_O_3_	Others
HAP	61.82	36.90	0.19	0.08	0.54	0.16	0.31
CAP	58.25	27.87	3.29	2.98	2.00	0.39	5.22

**Table 2:** The corresponding relationship between infrared absorption peaks and functional groups.

Vibration Type	Wavenumber (nm^−1^)		
	HAP	TiO_2_	ATO
Ti-O-Ti [14]	—	500–800	500–800
PO_4_^3−^ (γ_4_) [24]	567, 607	—	559, 601
CO_3_^2-^ [25]	874, 1417, 1452	—	874, 1417, 1452
PO_4_^3−^ (γ_1_) [26]	963	—	959
PO_4_^3−^ (γ_3_) [27]	1041, 1097	—	1037, 1094
H-O-H (HAP) [28]	1655	—	1654
Ti-OH (TiO_2_) [13]	—	3299	3275
P-OH (HAP) [29]	3569	—	3572

**Table 3:** Glaze properties of ATO-40, ATO-50, ATO-60 ceramic samples as opacifiers.

Opacifiers	L*	a*	b*	Melting Length (cm)	Gloss (GU)
ATO-40	74.10	0.76	1.76	4.82	92.3
ATO-50	89.99	−0.85	3.37	4.79	89.0
ATO-60	84.41	−0.40	3.85	4.69	90.5

**Table 4:** Glaze properties and characteristics of ceramic samples with different opacifiers (ATO, PTO, ZrSiO_4_, and TiO_2_) added.

Opacifies	L*	a*	b*	Color	Glaze Characteristics
ATO	89.99	−0.85	3.37	White	Smooth, delicate, bright
PTO	88.10	−1.07	3.10	White	
ZrSiO_4_	88.24	−0.02	2.29	White	
TiO_2_	92.13	−1.16	9.18	Yellow	

**Table 5:** EDS analysis results of the specified area of the glaze layer (ATO-50).

Elements	Ti	Si	Ca	O	Al	K	Totals
Mass percentage (%)	23.30	13.73	20.63	39.80	1.52	1.03	100.00
Atomic percentage (%)	11.98	12.04	12.68	61.27	1.38	0.65	100.00

## Data Availability

Data are contained within the article.
